# Occurrence and Risk Factors for Perioperative Treatment Discontinuation during Magnetic Resonance-Guided High-Intensity Focused Ultrasound (MR-HIFU) Therapy in Symptomatic Uterine Fibroids—A Retrospective Case–Control Study

**DOI:** 10.3390/jcm12185999

**Published:** 2023-09-16

**Authors:** Jakub Kociuba, Tomasz Łoziński, Kamil Latra, Lidia Korczyńska, Artur Skowyra, Elżbieta Zarychta, Michał Ciebiera

**Affiliations:** 1 Second Department of Obstetrics and Gynecology, Center of Postgraduate Medical Education, 00-189 Warsaw, Poland; lidka.korczynska@gmail.com (L.K.); skowyra.artur@gmail.com (A.S.); erhone@gmail.com (E.Z.); michal.ciebiera@gmail.com (M.C.); 2 Warsaw Institute of Women’s Health, 00-189 Warsaw, Poland; 3 Development and Research Center of Non-Invasive Therapies, Pro-Familia Hospital, 35-302 Rzeszów, Poland; tomasz.lozinski@pro-familia.pl; 4 Department of Obstetrics and Gynecology, Pro-Familia Hospital, 35-302 Rzeszów, Poland; latrakamil@wp.pl; 5 Department of Gynecology and Obstetrics, Institute of Medical Sciences, Medical College of Rzeszów University, 35-055 Rzeszów, Poland

**Keywords:** magnetic resonance-guided high-intensity focused ultrasound, uterine fibroid, leiomyoma, failure rate, treatment discontinuation

## Abstract

Background: The main aim of our study involves the analysis of reasons and risk factors for perioperative treatment discontinuation in patients with symptomatic uterine fibroids (UFs) who were qualified for magnetic resonance-guided high-intensity focused ultrasound (MR-HIFU) and in whom the procedure was discontinued. Methods: The presented research included 372 women who were primarily eligible for MR-HIFU, but the procedure was interrupted. The reasons and risk factors for treatment discontinuation were analyzed. A statistical comparison of two cohorts (patients in whom the treatment was discontinued and completed) was conducted based on epidemiological factors, UF characteristics and the implementation of uterotonics. Results: The mean discontinuation rate was 18.28% (*n* = 68). The main reason was the malposition of the intestines (52.94% of all cases). The thermoablation of subserosal UFs was a statistically significant risk factor of perioperative treatment discontinuation (OR 4.62, CI 95% 2.04–10.56), while the therapy of intramural UFs considerably decreased the risk (OR 0.21, CI 95% 0.08–0.51). The volume of the targeted UF was negatively correlated with the risk of discontinuation (OR 0.991, CI 95% 0.986–0.996). Augmentation with oxytocin, but not misoprostol, during the procedure significantly decreased the risk of potential discontinuation (OR 0.15, CI 95% 0.045–0.387, *p* < 0.001). Conclusion: Although the discontinuation rate seems to be relatively low, further prospective randomized trials are needed to confirm our results. The establishment of particular eligibility criteria for the treatment is a crucial issue in this area. Resigning from the procedure in cases at a high risk of discontinuation might increase patient safety and shorten the time to introduce the most appropriate therapy.

## 1. Introduction

Uterine fibroids (UFs) are the most common benign female genital tract lesions affecting women of reproductive age [1,2]. Their pathophysiology is complicated and not fully explained. Several mechanisms are proposed to explain the development and growth of UFs. Sex hormones, molecular changes in the extracellular matrix, growth factors, epigenetic and epitranscriptomic regulation and DNA damage repair pathways seem to play the most crucial role [3,4]. The problem of UFs is faced by up to 70% of women at menopausal age. Most UFs are asymptomatic, but approximately 25% of affected individuals have symptoms such as heavy menstrual bleeding, pelvic pressure, pain, and bowel or bladder dysfunction. Furthermore, UFs may be associated with impaired fertility, miscarriages or adverse pregnancy outcomes [1,5]. Numerous epidemiological risk factors increase the incidence of UFs. Black race, perimenopausal age, family history and obesity are proposed to be the most relevant ones [6].

Considering the high incidence, UFs are a serious socioeconomic problem for healthcare systems. The mean total all-cause cost may reach up to USD 16 792 per patient [7]. Researchers estimated the total costs can be as high as USD 5.9–34.4 billion each year in the United States [6].

A number of modalities are available in the therapy of UFs. As in most cases UFs do not cause any symptoms, the majority of patients require only clinical observation [8]. In the case of symptomatic patients, the therapy should be patient-tailored and adjusted to the patient’s preferences and expectations as much as possible [8]. Globally, the treatment can be divided into conservative and surgical modalities [8]. Conservative treatment concentrates mostly on pharmacological agents (e.g., non-steroidal anti-inflammatory drugs (NSAIDs), oral contraceptives, progestins and GnRH agonists/antagonists) [8,9,10]. Although the main aim of the modalities is to offer the patient symptom relief, for some of them adverse drug reactions and limited success rate may be unacceptable [1,11]. Conversely, surgical methods, such as hysterectomy or myomectomy, mostly resolve the cause of the symptoms, but they may be linked to long- and short-term complications and long post-operative recovery [12].

Nowadays, minimally invasive modalities, such as laparoscopic myomectomy, uterine artery embolization (UAE) or magnetic resonance- or ultrasound-guided high-intensity focused ultrasound (MR-HIFU/US-HIFU) provide a great opportunity for patients to reduce morbidity and mortality connected with open surgery, improve the quality of life by significantly reducing the symptoms of UFs, and decrease costs for healthcare systems [8,13]. According to currently available data, UAE and myomectomy seem to generate similar costs. However, MR-HIFU is expected to be even cheaper due to faster recovery. Research is still ongoing to confirm the above [14,15].

MR-HIFU is a radiological procedure using a concentrated focused ultrasound beam to obtain the thermal necrosis of the targeted tissue [16]. Magnetic resonance (MR) guidance enables planning the area of the treatment and facilitates real-time control of the temperature [17]. According to available data, this method seems to be a good alternative for surgical modalities, as the adverse events rate after treatment (especially severe ones) is relatively low and the efficacy of the therapy is satisfactory for the patients [18,19,20,21]. The recent meta-analysis from 2022 investigating the efficacy of the procedure established that the transformed symptom severity score (tSSS) in the Uterine Fibroid Symptom–Quality of Life (UFS-QoL) questionnaire declined after MR-HIFU on average by 54% in follow-up period [21]. It was similar to the results obtained after myomectomy or hysterectomy [22,23].

Although MR-HIFU is a good alternative to classic treatment methods of UFs, it may be offered to a selected group of patients. Since the United States Food and Drug Administration (FDA) approval in 2004 concerning the use of the MR-HIFU system (ExAblate 2000, InSightec, Ltd., Tirat Carmel, Israel) in UF therapy, this ablative technique has become more available in clinical practice. In 2009, the FDA modified the guidelines allowing for complete ablation to improve the outcomes while maintaining the same safety profile [24]. Moreover, not all UFs are suitable for thermal ablation [25].

Not all primarily qualified patients may complete the treatment. Good qualification and assessment of the feasibility of the procedure is the key matter in primary patient consulting. Regarding the potential costs of the treatment, it is worth considering if it is reasonable to begin the procedure in patients who will most likely not be able to complete the treatment. Proper assessment of eligibility criteria emphasizes patient-tailored therapy, which is one of the main issues in UF therapy.

All these arguments encouraged us to present our experience, since our center is one of the largest in central and eastern Europe and has a large cohort of patients with UFs treated with MR-HIFU.

Our case–control study aimed at the retrospective analysis of the causes and eligibility criteria of MR-HIFU therapy in patients who were primarily qualified for the procedure and failed to undergo the complete treatment at our reference center.

## 2. Materials and Methods

The study was conducted at the Department of Obstetrics and Gynecology of Pro-Familia Hospital in Rzeszów (Poland) in cooperation with the 2nd Department of Obstetrics and Gynecology of the Center of Postgraduate Medical Education (Warsaw, Poland). The research was approved by the Bioethics Committee (approval no 6/2022) at the Center of Postgraduate Medical Education, Warsaw, Poland and met the principles of the Declaration of Helsinki. The authors obtained written informed consent from all the patients. Data were retrospectively retrieved from medical registers.

A group of 1453 patients with symptomatic UFs was referred between April 2015 to May 2022 for MR-HIFU treatment. After clinical examination, 1178 of them were qualified for MRI screening for eligibility for MR-HIFU procedure. Finally, 372 patients were selected for the treatment and included in the study.

The inclusion criteria for the study were: the diagnosis of symptomatic UFs (heavy bleeding, painful intercourse, pelvic pressure, pregnancy-related complications such as miscarriages or preterm birth, and infertility, until 2018, and only with UFs as the only cause). We performed the treatment only in patients with UF types 1 and 2 according to Funaki [26]. In the years 2015–2018, the requirements of the scientific grant limited the inclusion criteria to women no older than 43. Afterwards, the age criterion was extended until 50 and patients with other causes of infertility were also included.

A part of the cohort received uterotonics (misoprostol with diclofenac or oxytocin) directly before the MR-HIFU procedure. The administration of those pharmacological agents was included in the protocol of our previous research. The inclusion, exclusion criteria and outcomes were described in detail in the former studies [27,28]. Those women were also included in our current research.

Patients with contraindications for MRI, large UFs (>13 cm), multiple UFs (>2), UFs located on the posterior wall of the uterus directly on the rectum, and pedunculated and asymptomatic UFs were excluded from the analysis. A history of operations due to UFs was a relative contraindication. Patients with Funaki type 3 UFs were also disqualified from the treatment and excluded from the analysis.

At the beginning of the qualification, all patients underwent a medical interview. They were asked to complete questionnaires regarding the symptoms of UFs. The next step involved clinical examination with special attention paid to the mobility and size of the uterus, and the position and location of UFs. All patients underwent a cervical smear to exclude a potential malignancy. Consecutively, transvaginal ultrasound with the assessment of the adnexa and uterus was performed by experienced sonographers with special attention to the size, location and position of UFs. 

After primary qualification, eligible patients were referred for MRI screening. The assessment of the pelvic area was performed with the administration of intravenous gadolinium contrast (Gadovist; Bayer Schering Pharma, Leverkusen, Germany) with T1- and T2-weighted scans before and after receiving the agent. UFs were divided according to the Funaki classification [26]. UFs that were hyperintense in T2-weighted images (Funaki type 3) were excluded. MRI evaluation also included the assessment of the size, volume, position and location of the uterus and UFs in relation to the uterine cavity and other pelvic organs such as the rectum, bladder and intestines. A possible acoustic window for thermoablation and requirement for intestinal mitigation was also assessed. Prior to the procedure, all patients were catheterized and we used mitigation techniques with rectal gel filling in all cases in which it was necessary (approximately 1/3 of the cases). Sonalleve integrated with Ingenia 3T MRI scanner (Philips, Amsterdam, the Netherlands) was the MR-HIFU device used for the treatment and primary MRI screening. After the treatment, all the patients who succeeded in undergoing the complete procedure were given an additional dose of gadolinium contrast in order to calculate the non-perfused volume ratio (NPVR) which was considered as the predictor of therapy success. 

In this case–control study, we would like to focus on the patients who failed to finish the treatment and the procedure was interrupted directly before or while it was in progress.

We selected the patients who did not complete the procedure despite primary qualifications. A comprehensive analysis of the causes of treatment discontinuation was performed. The next step involved dividing the whole cohort of 372 MR-HIFU patients into two groups: patients who underwent the complete treatment and individuals in whom the procedure was discontinued. We compared those two subgroups regarding the epidemiological factors (age, body mass index (BMI), parity), characteristics of the UFs (location, position, volume andFunaki type), the distance between the skin surface to UFs, and use of uterotonics (misoprostol with diclofenac and oxytocin).

The formal statistical analysis was conducted to assess the impact of the studied factors on the treatment discontinuation rate. We used the generalized linear logistic regression model and consecutively performed descriptive statistics with the Student’s t and χ^2^ test (depending on the character of a variable). The results of the univariate analysis of risk factors for treatment discontinuation were presented as odds ratios (OR) with a 95% confidence interval (CI). A *p*-value lower than 0.05 was considered statistically significant. 

## 3. Results

We performed MR-HIFU procedures in 372 patients during the analyzed time. The treatment was discontinued in 68 cases (18.28%), despite the positive primary qualification status. The malposition of the intestines, despite the use of mitigation techniques (bladder and rectal filling), was the main reason for perioperative discontinuation. It occurred in 36 cases (52.94%). The second most common reason for discontinuation was no reaction of the targeted UF to the sonication, i.e., no heating was observed despite maximal achievable power. It occurred in five patients (7.35%). Less common causes included the necrosis of a primarily eligible UF (2 cases, 2.94%), adenomyosis missed in the primary screening (1 case, 1.47%), and blood in the urine after beginning the procedure (1 case, 1.47%). In 1 case (1.47%) the treatment was interrupted due to pain. Due to the missing data, the reason for discontinuation could not be established in 21 patients (30.88%). All available data are presented in Table 1.

The comparison between the two subgroups (completed versus discontinued treatment) revealed that there were no statistically significant differences regarding the epidemiological data (age, BMI, and birth rate).

The characteristics of UFs were subsequently analyzed. The position of UFs seemed not to increase the risk of perioperative discontinuation of the procedure. No statistically significant differences occurred between both groups, regardless of the fact that the treated UF was on the posterior or anterior wall of the uterus.

As regards the location of the UFs in relation to the uterine cavity, we observed interesting significant differences. In the group in which the procedures were discontinued, we found that subserosal UFs were treated significantly more frequently (30% versus 8%, *p* < 0.001) and intramural UFs were treated less frequently (76% versus 93.9%, *p* = 0.001) compared to patients in whom the MR-HIFU procedure was completed. The risk of perioperative treatment discontinuation was more than 4-fold higher in the case of subserosal UF therapy (OR 4.62, CI 95% 2.04–10.56, *p* < 0.001), whereas the treatment of intramural UFs decreased the risk of discontinuation approximately 5 times (OR 0.21, CI 95% 0.08–0.51, *p* < 0.001). The prevalence of submucosal UFs was similar in both groups. The treatment of this type of UF seemed not to impact the discontinuation rate. The differences were not statistically significant.

The volume of the targeted UF was another factor that seemed to influence the risk of treatment discontinuation. Interestingly, we observed a significantly smaller volume of treated UFs in the group of discontinued procedures compared to the group of completed ones (48.15 mL versus 97.19 mL, *p* < 0.001). The risk of perioperative discontinuation increased with the decreasing volume of treated UFs (OR 0.991, CI 95% 0.986–0.996, *p* < 0.001).

Funaki UF type was a factor that did not seem to increase the risk of discontinuation of the procedure. The total incidence of both types (1 and 2) was similar and not statistically significant in both groups.

The distance from the skin surface to the targeted UF was another factor that we compared. Similar values were observed in both groups. The mean skin–UF distance in the discontinued procedure group was 97.11 mm, whereas in the group of completed procedures, it was 92.77 mm. The differences were not statistically significant.

The augmentation with uterotonics (misoprostol with diclofenac and oxytocin) was also compared in both groups. We observed a statistically significant lower rate of oxytocin use in patients in whom the treatment was discontinued compared to those who underwent the complete therapy (6% versus 29.3%, *p* < 0.001). The use of oxytocin during the procedure significantly decreased the risk of perioperative treatment discontinuation (OR 0.15, CI 95% 0.045–0.387, *p <* 0.001). Interestingly, this observation was absent in the case of misoprostol with diclofenac augmentation. The differences between both groups were similar (11.9% versus 19.4%, *p* = 0.21) and statistically insignificant. All the results are presented in Table 2 and Table 3.

## 4. Discussion

Although the treatment of UFs with MR-HIFU is a relatively new modality, the significance of this method in UF therapy is still increasing. Its widespread clinical use in UF treatment began in 2004 with the approval of the method by the US Food and Drug Administration (FDA) in this indication [29]. The eligibility criteria for the treatment have been changing for years. The first protocol accepted by the FDA included a number of limitations regarding UF characteristics (maximum 100 mL for one UF and 150 mL for 2 or more treated UFs, limitation of sonication up to 33% of UF volume) [30]. Since the method turned out to be quite safe and the experience of clinicians has grown, the eligibility criteria have been liberated. In 2019, Kröncke et al. published guidelines developed during a gynecological–radiological expert meeting regarding the MR-HIFU therapy of UFs [31]. This document contained the eligibility criteria for the treatment concerning the characteristics of UFs. The therapy was contraindicated in pedunculated subserosal UFs, submucosal type 0 and I according to the FIGO, more than 5 UFs, lesions positioned near the os sacrum, UFs with the diameter >10 cm (relative). However, the authors stated that the data concerning UF number and volume were unclear.

Although current research for proper eligibility criteria regarding the treatment and intraoperative failure rate is limited, some authors included this outcome in their research. A meta-analysis published by Verpalen et al. in 2019 investigated technical treatment failure rate based on 7 studies [24]. The rate of treatment failure was 3.5%. However, in some studies, the treatment failure rate was defined as no decrease in NPVR after the procedure [32]. The absence of a clear definition regarding treatment failure makes it difficult to draw final conclusions in this area. Some authors tried to predict treatment success regarding eligibility criteria. In 2020, Verpalen et al. reported a failure rate of 12.1%. In another paper, the same authors observed a decrease in treatment discontinuation rate from 40% to 12.8% after using a special manual manipulation protocol [20,33]. In their first study, the main reasons for intraoperative procedure discontinuation were inadequate heating (3.2%), the treatment of Funaki type 3 UF (2.4%), physical discomfort or pain (2.4%) and the interposition of the intestines (2.4%) [20]. The only evaluated factor that was found to influence the results was the Funaki type of UF. No statistically significant differences occurred between the treatment of Funaki type 1 and 2, while type 3 significantly increased the failure rate. The authors concluded that the discontinuation rate might depend on the experience of the radiologist and the malposition of the intestines. Therefore, no mitigation techniques were used. In the second study, apart from standard mitigation techniques such as bladder–rectal filling, the authors used a special uterine manipulation protocol and observed a reduction in the failure rate of 18% [33]. The highest reduction occurred in patients with the interposition of the intestines (20% versus 2.1%). This observation corresponds to our results, as the main reason for discontinuation despite bladder–rectal filling was the malposition of the intestines (52.94% of all incomplete procedures). Although these conclusions encourage an improvement in mitigation techniques to increase the eligibility for the treatment, the available data are somehow incoherent. Further randomized, prospective studies are needed to draw final conclusions and introduce a standardized mitigation protocol into clinical practice.

Regarding our data, the volume of the treated UF was a factor with the most significant influence on procedure discontinuation. As previously mentioned, the smaller the UF, the higher the risk of treatment discontinuation was (OR 0.991, CI 95% 0.986–0.996, *p* < 0.001). In the majority of currently available studies, the volume of treated UFs was not considered as an eligibility criterion [20,32,33,34,35,36,37,38,39]. In most cases, the eligibility criterion specified the maximal diameter of the lesion and, sometimes, also the minimal one. Most commonly, researchers indicated the minimal diameter criterion to be >3 cm [36,39,40,41,42,43,44,45]. Some authors also reported the treatment of UFs >1 cm [33]. Our data encourages further discussion to analyze whether the minimal volume of UFs should be considered as an inclusion criterion for the treatment. Moreover, in our opinion, it is worth considering whether the symptoms reported by patients with small UFs are exclusively connected with the presence of the lesions. Medical interviews and examinations seem to be crucial. In some cases, it may turn out that small UFs are in fact low symptomatic ones. Therefore, after presenting the potential risks, costs and benefits of the therapy, such patients may decide that clinical observation would be the best option for them. Nevertheless, further prospective randomized trials are needed to draw final conclusions in this area.

The location of the UF in relation to the uterine cavity also seems to play a role in treatment discontinuation. Our data showed that the therapy of subserosal UFs increased the risk for perioperative treatment discontinuation more than 4-fold (OR 4.62, CI 95% 2.04–10.56, *p* < 0.001). Although the paucity of data in this area limits the comparison of our results with those published by other authors, in some aspects they seem to have a correlation. Mindjuk et al. observed significantly lower NPVR in patients with a subserosal component of UFs [46]. The authors concluded also that bowel mitigation techniques were needed more frequently in the case of smaller UFs. Such observations are somehow coherent with our results. In the majority of currently available studies, the subserosal location of UFs was not considered as an exclusion criterion [20,32,33,34,35,36,37,38,39]. In our opinion, it is worth encouraging further discussion among researchers to analyze if small subserosal UFs are feasible for the procedure, even with the implementation of bowel mitigation techniques. Moreover, the therapy of small subserosal UFs should be carefully considered. In the case of small lesions, the symptoms reported by some women may be due to other causes and the most appropriate modality may involve clinical observation. If the patient is qualified for the treatment, healthcare professionals should inform her about potential benefits and risks (including an increased risk of failure) in this particular group of lesions.

The influence of the implementation of uterotonics on the treatment discontinuation rate is another interesting observation related to our analysis. We previously observed that the use of uterotonics (oxytocin and misoprostol) significantly improved the outcome of the procedure, especially in patients with highly vascularized UFs [27,28]. Our present research revealed a significant risk reduction for treatment discontinuation if oxytocin was administered during the procedure (OR 0.15, CI 95% 0.045–0.387, *p <* 0.001). Misoprostol has similar properties as oxytocin. According to our previous study, the outcomes of the procedure were significantly improved in both groups compared to the controls [28]. Such a correlation was not observed in the present study. Only patients who received oxytocin during the procedure had a significantly lower chance of treatment discontinuation. We suppose that the route of administration may play a role in the results. The transvaginal application of misoprostol could differ from the intravenous administration of oxytocin in pharmacokinetics. An immediate effect on uterine cells could be obtained at the beginning of the procedure in the case of oxytocin, while the transvaginal route of misoprostol application might be related to the longer absorption of the drug. A slower, but more stable increase in drug concentration may have affected the outcomes, but not influenced by the reduction of discontinuation rate. Nevertheless, this hypothesis needs to be confirmed by further studies.

Although our study revealed several interesting correlations, it also has some limitations.

Due to the missing data, we could not identify the cause of the treatment in 30.88% of cases. The design of a retrospective, case–control study and a change in patient selection criteria due to the scientific grant requirements may influence the risk of bias. However, the changed criteria regarded mostly the age and treatment of infertility, so we suppose the impact was not considerable. An influence of the increasing experience of the team who planned, qualified and conducted the procedure is another important issue. As the feasibility of achieving the acoustic window with mitigation techniques was assessed subjectively by the team members, it could have a slight influence on the obtained results. 

Despite the above-mentioned limitations, our research is characterized by several strengths. 

To the best of our knowledge, it is the first study analyzing the risk factors for perioperative treatment discontinuation in such a comprehensive way on such a large sample. Our study emphasizes the current trend in the scientific literature to make the therapy of UFs adjusted to patients’ expectations as much as possible. We hope the study findings may stimulate further discussion concerning the eligibility criteria for the treatment.

## 5. Conclusions

Perioperative treatment failure may be a serious clinical problem concerning both patients and healthcare professionals. Appropriate eligibility criteria for the treatment seem to play the most crucial role in patient consulting. In the future, adequate qualification may allow cost reduction for healthcare systems and a decrease in potential risks for the patient in case of unsuccessful treatment. 

Our data suggests that subserosal UFs with a small volume are connected with the highest risk of perioperative treatment discontinuation. In the case of augmentation with uterotonics, the intravenous administration of oxytocin (but not transvaginal application of misoprostol/diclofenac) during the procedure significantly decreases the risk of treatment interruption.

Although the discontinuation rate seems to be relatively low, further prospective, randomized trials are needed to establish eligibility criteria for the treatment. This would allow clinicians and patients to make more informed decisions regarding this type of therapy.

## Figures and Tables

**Table 1:** Causes of perioperative treatment discontinuation.

Cause of MR-HIFU Discontinuation	[*n*]	[%]
Malposition of intestines	36	52.94
No reaction to sonication	5	7.35
Necrosis of the UF	2	2.94
Pain	1	1.47
Blood in urine	1	1.47
Adenomyosis	1	1.47
Not available	21	30.88
Total	68	100

**Table 2:** Comparison between patients in whom the procedure was completed and discontinued.

Characteristics	Procedure Discontinued			Procedure Completed			*p*-Value
	Total	Mean	SD	Total	Mean	SD	
Age (y)	68	35.41	19.77	304	36.65	5.32	0.103
BMI (kg/m^2^)	68	22.96	4.95	285	23.29	3.67	0.496
Distance from skin surface to UF (mm)	66	97.11	22.6	297	92.77	21.36	0.141
Fibroid volume (mL)	67	48.15	55.85	291	97.19	104.22	<0.001 *
	Total	Number of Patients	Procedure Discontinued [%]	Total	Number of Patients	Procedure Completed [%]	*p*-Value
Birth rate	59			282			0.675
0		41	69.5		187	66.3	
1		11	18.6		64	22.7	
2		7	11.9		27	9.6	
3		0	0		4	1.4	
Position of UF	51			192			
on the anterior wall		33	64.7		137	71.4	0.454
on the posterior wall		19	37.3		59	30.7	0.472
Location of UF	50			165			
subserosal		15	30		14	8.5	<0.001 *
submucosal		2	4		14	8.5	0.453
intramural		38	76		155	93.9	0.001 *
UF modeling the uterine cavity	47	32	68.1	183	135	73.8	0.551
Funaki II	66	24	36.4	295	121	41.6	0.522
Misoprostol use	67	8	11.9	268	52	19.5	0.212
Oxytocin use	67	4	6	266	78	29.3	<0.001 *

SD—standard deviation, BMI—body mass index, UF—uterine fibroid, *—statistical significance.

**Table 3:** Univariate analysis for the risk of treatment discontinuation.

Characteristics	OR	CI 2.5%	97.50%	*p*-Value
Age	0.959	0.913	1.009	0.104
BMI	0.974	0.901	1.048	0.495
Birth rate	0.929	0.609	1.363	0.718
Fibroid volume	0.991	0.986	0.996	<0.001 *
Distance from skin surface to UF	1.009	0.997	1.022	0.141
UF on the anterior wall	0.736	0.386	1.436	0.358
UF on the posterior wall	1.338	0.694	2.535	0.376
Subserosal UF	4.622	2.043	10.57	<0.001 *
Submucosal UF	4.622	0.069	1.685	0.301
Intramural UF	0.204	0.08	0.507	<0.001 *
UF modeling the uterine cavity	0.759	0.383	1.552	0.436
Funaki II	0.803	0.457	1.386	0.436
Misoprostol use	0.563	0.237	1.191	0.159
Oxytocin use	0.153	0.045	0.387	<0.001 *

OR—odds ratio, CI—confidence interval UF—uterine fibroid, BMI—body mass index, *—statistical significance.

## Data Availability

The data used to support the findings of this study are available from the corresponding author upon request.
